# Tricuspid Regurgitation in Acute Heart Failure: Predicting Outcome Using Novel Quantitative Echocardiography Techniques

**DOI:** 10.3390/diagnostics13010109

**Published:** 2022-12-29

**Authors:** Max Berrill, Eshan Ashcroft, David Fluck, Isaac John, Ian Beeton, Pankaj Sharma, Aigul Baltabaeva

**Affiliations:** 1 Department of Cardiology, St. Peter’s Hospital, Chertsey KT16 0PZ, UK; 2 Department of Research and Development, St. Peter’s Hospital, Chertsey KT16 0PZ, UK; 3 Institute of Cardiovascular Research, Royal Holloway University, UCL, Egham TW20 0EX, UK; 4 Department of Cardiology, Royal Brompton & Harefield Hospital, London UB9 6JH, UK

**Keywords:** acute heart failure, tricuspid regurgitation, disproportionate TR

## Abstract

Background: The prognostic impact of tricuspid regurgitation (TR) in acute heart failure (AHF) remains uncertain. Methods: We retrospectively assessed 418 consecutive AHF patients who underwent comprehensive echocardiographic assessment within 24 h of study recruitment. TR was quantitatively assessed with 3 guideline-directed measures: regurgitant volumes (RgVol), effective regurgitant orifice area (ERO) and vena contracta (VC) diameter. Disproportionate TR was assessed by the ratio of the VC diameter to the tricuspid annulus diameter (VC/TA) ≥ 0.24. Results: The prevalence of significant (i.e., >mild) TR differed when various standard assessment parameters were applied to quantification: RgVol 50.3% (173/344), ERO 75.6% (260/344) and VC diameter 94.6% (335/354). None were able to delineate those at excess risk of all-cause 2-year mortality using guideline-directed cut-offs of mild, moderate and severe TR. Using a cut-off of VC/TA ≥ 0.24, we identified that 36.9% (130/352) had “disproportionate” TR. Disproportionate TR was associated with an excess risk of mortality at 2 years compared to proportionate TR; HR 1.48 (95% CI 1.06–2.06 [*p* = 0.02]) which was not significant on multivariate assessment (*p* = 0.94). Conclusions: TR was not associated with outcome in AHF using guideline measures. A new assessment of “Disproportionate” TR carries a higher risk than proportionate TR but was not related to outcome based on multivariate analysis. Further research is needed to quantify TR more effectively to identify cut-offs for future guidelines and disproportionate TR may be an important part of Heart Failure 2.0.

## 1. Introduction

Acute heart failure (AHF) remains a substantial cause of morbidity and mortality. It accounts for a quarter of European in-hospital deaths or discharges [1,2] and in the United Kingdom acute heart failure admissions are associated with a 9.3% in-hospital mortality [3]. Consequently, novel diagnostic markers for risk stratification are an important part of current research.

Tricuspid regurgitation (TR) is present in 60% of hospitalized AHF patients [1,2], the vast majority is functional [4] and half is greater than mild severity [1,2]. Despite this high prevalence, the prognostic impact of TR in AHF remains highly complex [5,6,7,8,9].

This uncertainty is a consequence of a diverse array of comorbid conditions straining the right heart alongside the technical challenges of precise quantitative assessment of the tricuspid valve apparatus and the right ventricle (RV) [10].

Unlike left heart geometry, there is substantial difficulty obtaining orthogonal views of the RV and the tricuspid valve. Significant variation in RV/right atrial/tricuspid annular remodelling makes linear assessments (i.e., vena contracta (VC) diameters and PISA radii) highly variable between individuals and between imaging planes within the same individual [11,12].

International guidelines rely on a mixture of qualitative, semi-quantitative and quantitative assessments [13,14] but there is inconsistency in their application within clinical studies. For example, in a large AHF study of 639 individuals, quantitative assessment relied solely on apical 4 chamber VC diameters and tenting area [4]. In another prospective study which enrolled 1824 patients, PISA estimates were available in <5% of patients [6]. These inadequacies predicate the need for more novel assessments of TR.

A novel concept of disproportionality has been developed in the context of MR in chronic HF [15,16,17], which we have also applied to AHF [18]. We feel this is a promising novel diagnostic concept to be applied in the acute setting. This concept has been applied to TR in a retrospective analysis, suggesting that “disproportionate TR” was associated with an excess risk of mortality (HR 1.57) at 5 years in patients with moderate and severe TR and preserved ejection fraction [19]. A cut-off of ≥0.24 (i.e., 24%) was used to identify what we have termed here “disproportionate TR” by indexing the VC diameter to tricuspid annulus diameter. However, the effect of disproportionate TR on outcomes has not been assessed within the context of *acute* heart failure.

## 2. Materials and Methods

This was a retrospective analysis to assess for the impact of TR in AHF using the Mitral Regurgitation in Acute Heart Failure (MRAHF) study results. MRAHF study details are published previously [18]. In summary, this was a prospective, observational study over 12 months of 418 individuals presenting in AHF who were included with raised brain-natriuretic peptide (BNP) level (>100 pg/mL) and evidence of heart failure on comprehensive echocardiographic assessment. Echocardiography occurred within 24 h of recruitment to the study. Patients with severe infection, respiratory failure secondary to pulmonary causes and those with chronic heart failure admitted with a non-AHF diagnosis were excluded.

Participants were followed up for 2 years and assessed for all-cause mortality through the UK summary care record system used nationally by general practices (community healthcare practices) in the United Kingdom and by the online software Evolve^TM^ (Kainos, Burnley, UK) for patient records, including death certificates, used at our centre.

Trial oversight was by the Ashford & St Peter’s NHS Trust Research and Development team and was approved by the institutional review board and ethics committee. All patients gave written informed consent prior to enrolment in the study. All authors had access to data and this manuscript for review. The experimental design and decision for publication was by AB. Most statistical analysis was carried out at an independent company with established expertise in medical trials with input from MB.

### 2.1. Echocardiography

Echocardiography was carried out with G.E. Vivid S70 (GE Healthcare, Chicago, IL, USA) and analysed and stored using EchoPac v202.5 (GE Healthcare, USA). Most exams were performed by a single accredited operator with a dedicated protocol (Supplementary File S1). Offline measurements were carried out by two experienced echocardiographers (both with >5 years echo experience and both with British Society of Echocardiography Transthoracic Echocardiography Level II accreditation). A sample of these were cross-referenced by a consultant cardiologist with an expertise in echocardiography (>20 years practice as a cardiology imaging expert) who is a Fellow of the European Society of Cardiology and clinical lead at the Department of Echocardiography at Harefield Hospital—a high-volume cardiothoracic surgical, transplant and tertiary referral centre with European Association of Cardiovascular Imaging accreditation.

Assessment of left and right atrial and ventricular geometry, systolic and diastolic function were obtained using a standard TTE minimum dataset approach advocated by the British Society of Echocardiography [20]. The LV tissue Doppler imaging S wave velocity (LV S’) was taken from the lateral mitral annulus in the apical 4 chamber view. The LV dP/dt was estimated from Doppler continuous wave (CW) interrogation of the mitral regurgitant jet calculating the slope between 1 m/s and 3 m/s, with optimized scale and sweep-speed. RV systolic assessments of fractional area change (RVFAC), S’ and tricuspid annular systolic excursion (TAPSE) were measured from the RV-focussed apical 4-chamber view.

Traditional TR assessment was performed by qualitative, semi-quantitative and quantitative parameters to gauge lesion severity according to the 2017 guidelines of the American Society of Echocardiography Society and society for Cardiovascular Magnetic Resonance [13]. Quantitative parameters assessed were regurgitant volume (RgVol), effective regurgitant orifice area (ERO) and VC diameter. ERO and RgVol were calculated using the flow convergence method, with the largest flow convergence zone selected visually. A majority method for quantitative assessment was used where 2 of 3 features needed to be present to categorize a lesion into the appropriate, corresponding severity group. 

Tricuspid annulus and VC diameter used in the disproportionate assessment of TR were obtained from the RV-focussed apical four chamber view. The vena contracta was selected visually from the best image in mid-systole. The TA diameter measurement was made from the frame closest to end-systole where the leading-edge to leading-edge diameter was at its widest by visual assessment. In the interest of image quality and optimal frame rate for accurate and precise measurement, a different image from the same acoustic window and probe position was used for vena contracta. Disproportionate TR was defined by the ratio of the VC diameter to the tricuspid annulus diameter, with a binary threshold of ≥0.24 used to identify disproportionate TR. This cut-off was previously identified by Fortuni et al. [19].

RV GLS free wall analysis was performed using an optimized RV apical 4 chamber view. One cardiac cycle was isolated where optimal visualisation of the free wall myocardium was obtained. A region of interest (ROI) was defined segmentally from free wall base to the apex. This was performed as a ‘single wall’ analysis excluding the interventricular septum to minimize the impact of LV myocardial deformation. GE EchoPAC LV 2D strain package was repurposed for this retrospective analysis of RV strain.

RV peak GLS was defined as the maximal strain value obtained at any point during systole. RV end-systolic GLS was defined by the strain value at the time of pulmonary valve closure (PVC). The timing of PVC was identified in the parasternal short axis view with Doppler spike of PVC on Doppler contour when the sample volume is placed immediately proximal to the pulmonary valve. GE EchoPac then automatically applies the corresponding timepoint on the ECG to identify PVC in the apical view used to analyse the RV strain outlined above. 

### 2.2. Statistical Analysis

Demographic and clinical cardiac characteristics were summarized for all participants at the point of study inclusion. Patients were divided into two groups according to the VC/TA cut-off ratio of 0.24. Between groups comparisons were carried out using non-parametric Mann–Whitney U test for continuous data (represented as median (inter-quartile range)), as normality testing (via Shapiro–Wilk) indicates data were not normally distributed, and chi-square test for categorical data (represented as %). Kaplan–Meier overall survival estimates were generated for four metrics of interest (TR RegVol, TR ERO, TR VCD, and VC/TA) with groupings being categorized as mild, moderate, and severe based on pre-determined guideline-directed thresholds for each variable. Univariate analyses were initially undertaken to identify potential associations with 2-year all-cause mortality. Significance was assessed using the log-rank method and Cox’s proportional hazards model. Individual associations with outcome were then included in a multivariate model. Statistical significance was accepted at a two-tailed α-value of 0.05 throughout.

Variables to be included in multivariate analysis were selected by MB & AB and included: age, gender, BMI, the presence of coronary artery disease, hypertension, diabetes, chronic kidney disease (CKD), chronic obstructive pulmonary disorder (COPD), previous cerebrovascular accident (CVA), right atrial size, left atrial size, moderate or greater MR, systolic pulmonary artery pressure, tricuspid annular plane systolic excursion (TAPSE), LV ejection fraction (LVEF), LV end-diastolic volume (LVEDV), blood urea nitrogen, plasma sodium, brain-natriuretic peptide (BNP), haemoglobin (Hb), estimated glomerular filtration rate (eGFR), RV end-diastolic area indexed to body surface area (RVA diastole index) and the presence or absence of disproportionate TR.

## 3. Results

616 patients presenting with signs of AHF were assessed for eligibility and 72.6% (447) of participants were recruited. 418 individuals were included in final analysis after excluding rehospitalisation (Supplementary File S2). Every patient had some form (i.e., at least “trace”) TR. 86.7% (363/418) had sufficient TR jet for reliable quantification. 4 patients were lost to follow-up.

Among those with 2-year outcome data: quantification of RgVol was available in 83.1% (344/414); 49.7% (171/344) displayed volumetrically defined mild TR, 12.5% (43/344) displayed moderate TR and 37.7% (130/344) severe TR. ERO assessment was available in 83.1% (344/414), with 24.4% (84/344) displaying mild ERO-defined TR, 26.4% (91/344) moderate and 49.1% (169/344) severe TR. VC diameters were available in 85.5% (354/414) of jets, 5.4% (19/354) displayed VC diameter-defined mild TR, 43.5% (154/354) moderate TR and 51.1% (181/354) severe TR. Disproportionate TR was identified in 36.9% (130/352) of patients with both VC and TA diameters available and proportionate TR in 63.1% (222/352).

Comparison of demographics, clinical background, presenting features and mortality are displayed of patients with and without disproportionate TR in Table 1. Patients with disproportionate TR were older and had a higher prevalence of previous cerebrovascular accidents but otherwise displayed a similar cardiovascular risk profile. BNP was also higher in disproportionate TR compared to proportionate TR (1074 pg/mL vs. 901 pg/mL [*p* = 0.034]), with lower eGFR (48.5 mL/m^2^ vs. 54 mL/m^2^). 2-year mortality was significantly higher in disproportionate TR (47.7% vs. 36.5% [*p* = 0.02]).

In disproportionate TR there were higher RgVol (61.0 mL vs. 17.0 [*p* < 0.001]), ERO (1.10 cm^2^ vs. 0.50 cm^2^ [*p* < 0.001]) and VC diameter (0.6 cm vs. 0.2 cm [*p* < 0.001]) (Table 2). Both the right atrium and the right ventricle were larger in disproportionate TR (all assessments *p* < 0.003). Both disproportionate and proportionate TR displayed similarly impaired (or borderline-impaired) longitudinal and radial systolic function measured by TAPSE (1.4 cm vs. 1.5 cm [*p* = 0.16] and RV FAC (37.9 vs. 33.7 [*p* = 0.16] (Table 2). Despite significant differences in values both groups displayed preserved S’ velocities (0.10 cm/s vs. 0.11 cm/s [*p* = 0.0023]) (Table 2). Systolic pulmonary artery pressures were also higher in disproportionate TR than proportionate TR (59 mmHg vs. 49 mmHg [*p* < 0.001]) (Table 2). RV strain was similar between groups.

The left atria were larger in disproportionate vs. proportionate TR (29.3 cm^2^ vs. 27.5 cm^2^ [*p* = 0.015]) but LV volumes were similar and within the normal range (Table 3). LVEF was similar between groups (43.0% vs. 45.0% [*p* = 0.63]) as were all other parameters of systolic function including LV strain and strain rate. Patients with disproportionate TR had a greater prevalence of more than mild MR (51.5% vs. 36.6 & [*p* = 0.0059].

**Figure 1:**
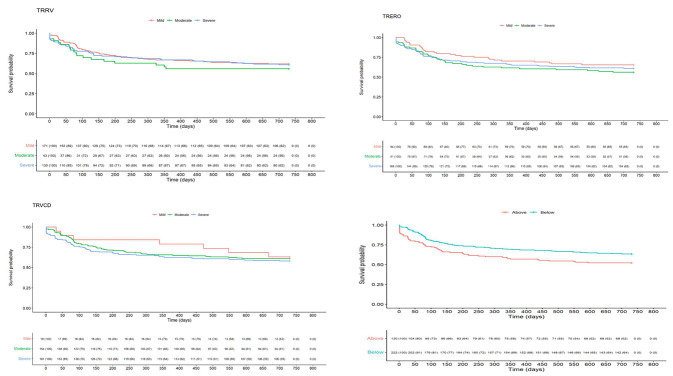
Unstratified Kaplan–Meier curves of 4 different assessment of tricuspid regurgitation (TR) severity and their impact on 2-year all-cause mortality with numbers at risk outlined beneath each panel. Clockwise from top-left: TR regurgitant volume (TRRV), TR effective regurgitant orifice (TRERO), TR vena contract to tricuspid annulus diameter (VC/TA) where curves display population at risk above and below a threshold of 0.24 and TR vena contract diameter (TRVCD).

Quantitative evaluation of TR by RgVol, ERO or VC diameter, delineated by guideline-directed cut-offs was unable to identify patients at risk of excess mortality at 2 years (Table 4 & Figure 1) [all *p* > 0.40]. When comparing >mild TR (i.e., haemodynamically significant TR) with mild TR, there were also no differences in outcome (Supplementary Table S1). When further assessing TR by severe vs. “non-severe” (i.e., ≤ moderate in severity) this could also not identify those at risk of poor outcome (Supplementary Table S2).

Disproportionate TR, defined by VC/TA ≥ 0.24 was associated with an excess risk of mortality (HR 1.48 (1.07–2.06) [*p* = 0.020]) (Table 5 and Figure 1). Other variables associated with increased risk of poor outcome identified on univariate analysis are displayed in Supplementary Table S3. However, in multivariate analysis of all patients, disproportionate TR was not independently associated with 2-year mortality (*p* = 0.95). Independent prognostic indicators associated with increased risk of mortality were age, the presence of COPD, LVEDV, blood urea and BNP (Table 6). 

59/414 patients with 2-year follow up met the criteria of a LVEF > 55% and >mild TR (i.e., a similar cohort to Fortuni et al.). Within this group, disproportionate TR was not significantly associated with worse outcome; HR 2.03 (95% CI 0.87–4.75 [*p* = 0.10]) (Supplementary Table S4). The results of multivariate analysis on this cohort, which did not include disproportionate TR, are displayed in Supplementary Table S5.

Data were analysed using a cox-proportional hazards model to generate survival estimates between subgroups of each variable and with respect to the covariates selected. The covariates listed were selected for post hoc, multivariate analysis if demonstrating statistical significance in the univariate analysis reported in Table 1.

## 4. Discussion

When undergoing early echocardiography, TR was a highly prevalent feature of those admitted with AHF with more than 85% displaying quantifiable regurgitation. The prevalence of TR severity varied depending on the quantitative feature used as a cut-off. TR severity defined by RgVol, ERO or VC diameter was unable to identify those at risk of poor outcome at 2 years, based on unadjusted Kaplan–Meier estimates. A novel assessment of disproportionate TR was not significantly associated with outcome on multivariate assessment.

These findings highlight the inconsistencies and challenges facing objective and quantitative assessment of TR. The guideline indicated assessments (RgVol, ERO and VC diameter) in this study described very different proportions of >mild TR (50.3%, 75.6% and 94.6%**).** These challenges are driven by several well-characterized limitations to the assessment of TR.

The lower pressure and higher dynamic respiratory variation within the right heart create greater loading-dependent variability of TR severity [13], making it challenging to consistently characterize regurgitant flows. These physiological dynamics significantly vary depending on the timing of haemodynamic assessment during admission and associated diuresis.

In MR, variation in severity with loading conditions or exercise is well characterized [21,22]. However, we are unaware of any assessment of the prevalence or prognostic significance of “dynamic TR” or TR that varies with loading conditions. We think the timing of echo assessment is likely critical in the evaluation of atrioventricular regurgitation in AHF. Currently, there is no consensus as to the optimum time for echocardiographic assessment in AHF.

We discovered at least mild TR in ~85% of our AHF cohort. This likely reflects the acute timing of echo assessment during this investigation with most patients assessed within 24 h into admission, prior to intensive diuresis. This may account for the higher proportion of patients identified with any form of TR compared to the 62.6% in the EuroHF study [1,2], which does not report on the average timing of echo assessment.

It is also often difficult to consistently measure VC diameters in orthogonal views. Ideally VC diameter should be interrogated to assess for a biplane width [23] (usually zoomed on the parasternal RV inflow and apical 4 chamber view [13]) with multi-view averages suggested for elliptical orifices [24].

Vena contracta assessment of significant TR is also limited in its capacity to stratify severity other than severe (i.e., >0.7 cm) [25] despite its inclusion in guidelines to delineate mild and moderate groups [13,26] which we have used in this study. Limitations of ERO and PISA include assumptions of the hemispherical shape of effective orifice, difficulties assessing multiple and/or eccentric jets and that velocity–time integrals and PISA heads are obtained in different cardiac cycles [13].

In this study a novel assessment of TR in which the VC diameter is indexed TA diameter, identified an excess risk of mortality (HR 1.48) in the 129/358 (36.0%) of patients with disproportionate TR, compared to proportionate TR. This, however, was not significant on multivariate assessment in our AHF cohort. Instead, drivers of outcome were confounders with known associations with outcome (age, admission urea, LVEDV, BNP, etc.). may reflect a more diverse comorbid profile than the original derivation study in Fortuni et al. Our entire MRAHF study cohort differed to Fortuni as they were older (78 yr vs. 70 yr), had a higher burden of cardiovascular risk factors and higher proportions of “haemodynamically significant” (i.e., >mild) MR (39% vs. 18%) [18]. Unlike Fortuni et al. we have assessed the role of disproportionate TR in those with guideline-suggested “mild” TR (provided it was quantifiable) given the wide discrepancy of standard parameters. Importantly in our cohort, 13.6% (18/132) of patients with disproportionate TR had RegVol within the mild range.

Furthermore, the impact of the same volume loading from TR could have different consequences depending on RV dimensions and compliance. In our cohort the difference in absolute RV end-diastolic areas (RVAd) was relatively smaller than the difference in TR volumes in the group of patients with disproportionate TR. The median RVAd indexed to BSA of both proportionate (10.1 cm^2^/m^2^) and disproportionate TR (12.2 cm^2^/m^2^) groups were within the normal range (<12.6 cm^2^/m^2^) [27]. By standard chamber quantification these would not be considered as remodelled right ventricular chambers. This replicated our previous findings on left ventricular volumes in presence of dysproprotionate MR in AHF [18]. These broadly normal end-diastolic ventricular dimensions suggest that major ventricular remodelling does not always happen in haemodynamically significant functional MR and TR.

It is interesting that a proportion (14.1%) of the total number of patients in our study met similar echo criteria to Fortuni (n = 59) study. In this subgroup there was a trend towards worse outcome (HR 2.03 95% CI 0.87–4.75 [*p* = 0.10]) but did not reach significance. Nevertheless, the presence of ‘Fortuni’ cohort in our study reflects on general applicability of the original Fortuni assessment to a “real-world” AHF setting given that original cohort data collection occurred over 21 years and was focused on stable patients with normal LV function. It will be interesting to collate the data from other AHF studies as it appears to show some utility when applied to the much broader AHF “all-comers” in this investigation.

Our data demonstrated that standard TR assessment cannot identify those at risk of poor outcome at all. Disproportionate TR meanwhile is not associated with outcome in isolation but, taken together with other factors, contributes to poor outcome and is a relevant non-invasive assessment of cardiac performance useful for frontline clinicians. Any potential role of disproportionate TR in future guidelines should be placed in the context that in both the Fortuni derivation cohort and our own AHF study, none of the guideline directed quantitative assessments of TR displayed a discriminatory capacity with respect to long-term outcome.

The novel assessment outlined by Fortuni et al. falls more broadly into an approach to the assessment of functional TR which places severity within the context of tricuspid annular diameter, mechanisms of tricuspid coaptation and tethering forces [28]. These changes are often a reflection of right ventricular/atrial dysfunction and remodelling. This attempt to place the severity of atrioventricular regurgitation within the context of atrial, annular and ventricular parameters is mirrored in more novel evaluation of MR, in which particular focus has been given to the concept of “disproportionate MR” [15,16].

This disproportionality framework continues to be debated, with doubt as to the relevance of indexing markers of mitral, or tricuspid, regurgitation to ventricular parameters [17]. This novel conceptual framework has been derived from conflicting results of two transcatheter randomized, controlled trials in functional MR [15], but doubt has been cast as to the haemodynamic plausibility of the echo data provided with many trial patients’ calculated cardiac index <2.2 L/min/m^2^ (i.e., near cardiogenic shock) [17].

There are also limitations to PISA-derived quantification of regurgitant pathology in general, with many assumptions on single, central or symmetrical jets which are captured on only one cardiac cycle, which is independent of the CW interrogated cardiac cycle for velocity time integral assessment. However, when these techniques are applied consistently by observers this should limit the inherent physiological shortcomings of Doppler assessment of regurgitant lesions.

For future work on disproportionate TR, there is an obvious discrepancy between the evaluation of disproportionate TR and MR because LV volumetric assessment is readily available in MR due to orthogonal views of left heart geometry. Attempts of RV volumetric assessments by echocardiography [29] have been validated in healthy individuals [30] and in acute heart failure [31] and offer a promising avenue for further research. Future assessments of either standard TR assessment or more novel indicators which index to right heart parameters will also need to reflect the ongoing consideration of more novel grading systems to reflect the wider spectrum of TR severity [32,33,34].

There are several limitations of this study. This was a single-centre study which limits the generalisability of its findings. A further limitation is that this is a retrospective analysis of a study designed to evaluate the impact of MR in AHF. Prospective studies to assess for TR, for example with prespecified to further investigate the prognostic impact of disproportionate TR are warranted.

In summary, this study highlights the variation in the quantitative evaluation of TR and inadequate discriminatory capacity to identify those at risk of long-term poor outcomes following hospitalisation for acute heart failure. It also highlights a role for more novel assessments of TR, including the evaluation of disproportionate TR, which warrant further study.

## 5. Conclusions

In summary, this study highlights the variation in the quantitative evaluation of TR and inadequate discriminatory capacity to identify those at risk of long-term poor outcomes following hospitalisation for acute heart failure. It also highlights a role for more novel diagnostic assessments of TR, including the evaluation of disproportionate TR, which warrant further study.

## Figures and Tables

**Table 1:** Baseline demographics, clinical history, presenting features and outcome of patients delineated by threshold for disproportionate TR.

	Tricuspid Regurgitation		
	Proportionate TR (VC/TA < 0.24) n = 224	Disproportionate TR (VC/TA ≥ 0.24) n = 132	*p* Value
Demographics			
Age (years)	81.0 (72.0–86.0)	83.0 (76.0–88.0)	0.0037
BMI (Kg/m^2^)	27.7 (23.2–32.0)	26.4 (22.6–30.9)	0.21
Gender (female %)	45.0	53.8	0.090
Clinical background			
Coronary artery disease (%)	38.8	34.1	0.37
Hypertension (%)	55.4	57.6	0.68
Diabetes (%)	32.1	25.8	0.20
Chronic Kidney Disease (%)	43.3	52.3	0.10
COPD (%)	14.3	18.2	0.33
Previous CVA (%)	13.4	22.7	0.023
Presenting features			
eGFR (mL/m^2^)	54.0 (38.5–61.0)	48.5 (33.0–61.0)	0.027
Haemoglobin (g/L)	122 (107–137)	121 (108–136)	0.85
CRP (mg/L)	14.0 (4.10–35.5)	16.5 (7.50–36.0)	0.20
BNP (pg/L)	901 (494–1586)	1074 (603–2008)	0.034
Systolic BP (mmHg)	135 (120–155)	130 (114–150)	0.10
Diastolic BP (mmHg)	73.0 (62.0–85.0)	72.5 (61.8–86.3)	0.81
Sinus rhythm (%)	40.9	32.3	0.11
Atrial Fibrillation (%)	45.0	53.8	0.11
Outcome			
2-year mortality (%)	36.5	47.7	0.032

Abbreviations: BMI (Body Mass Index). COPD (Chronic obstructive pulmonary disorder). CVA (Cerebrovascular Accident). eGFR (estimated Glomerular Filtration Rate). CRP (C-reactive Protein). BNP (Brain Natriuretic Peptide) Continuous variables are expressed as medians (interquartile range) with between-group differences evaluated using the Mann–Whitney U test. Proportions were compared using the chi-squared test. Patient data were missing for BMI (above: n = 1), eGFR (below: n = 1), CRP (below: n = 18, above: n = 9), Mortality (below: n = 2, above: n = 2) and GLSR (below: n = 2, above: n = 1).

**Table 2:** Right heart assessment of patients delineated by threshold disproportionate TR.

	Tricuspid Regurgitation		
Right Heart Parameters	Proportionate TR (VC/TA < 0.24 mL)	Disproportionate TR (VC/TA ≥ 0.24 mL)	*p* Value
Right atrium	n = 224	n = 132	
RA size (cm^2^)	22.4 (17.6–27.6)	25.2 (21.2–32.2)	<0.001
Indexed RA size (cm^2^/m^2^)	14.1 (11.5–18.3)	11.6 (9.55–13.9)	<0.001
Right ventricle size			
RV end-diastolic (cm^2^)	19.4 (16.0–24.5)	21.8 (17.5–28.4)	0.0027
Indexed RV end-diastolic area (cm^2^/m^2^)	10.1 (8.34–12.5)	12.2 (9.80–15.3)	<0.001
RV end-systolic area (cm^2^)	11.7 (8.75–16.2)	13.5 (10.3–18.9)	0.0015
Indexed RV end-systolic area (cm^2^/m^2^)	6.27 (4.58–8,49)	7.65 (5.49–10.6)	<0.001
TV annulus (cm)	3.50 (3.00–4.00)	3.60 (3.08–4.10)	0.55
Tricuspid regurgitation			
Regurgitant volume (mL)	17.0 (8.00–32.0)	61.0 (44.0–83.0)	<0.001
ERO (cm^2^)	0.20 (0.10–0.40)	0.60 (0.40–0.80)	<0.001
VC diameter (cm)	0.50 (0.30–0.60)	1.10 (0.90–1.32)	<0.001
Right ventricle performance			
RV FAC (%)	37.9 (28.8–46.5)	33.7 (25.6–45.0)	0.16
TAPSE (cm)	1.50 (1.12–1.80)	1.40 (1.10–1.80)	0.16
RV’S (cm/s)	0.11 (0.09–0.14)	0.10 (0.08–0.13)	0.0023
Right ventricular strain			
GLS free wall (peak) (%)	−13.8 (−17.2–−10.3)	−12.8(−17.5–−9.66)	0.23
GLS free wall (end systole) (%)	−12.8 (−16.3–−9.43)	−12.2(−16.7–−8.45)	0.50
Systolic pulmonary artery pressure (mmHg)	49.0(38.3–60.8)	59.0(47.3–70.0)	<0.001

All indexed parameters are divided by body surface area (m^2^). Abbreviations; Tricuspid regurgitation (TR), vena contracta/tricuspid annulus (VC/TA), right atrium (RA), right ventricle (RV), fractional area change (FAC), tricuspid annular plane systolic excursion (TAPSE), global longitudinal strain (GLS), effective regurgitant orifice (ERO). All data are presented as median (IQR) and between group differences were evaluated using the non-parametric Mann–Whitney U test. Patient data were missing for the following parameters (below/above threshold): RA size (below: n = 2), RV area (diastole) (below: n = 1, above: n = 1), RV area (systole) (below: n = 1, above: n = 1), RV FAC (below: n = 1, above: n = 2), TAPSE (below: n = 2), RV’S (below: n = 7, above: n = 4), GLSpeak (below: n = 31, above: n = 12), GLSend (below: n = 31, above: n = 12), Systolic pulmonary artery (below: n = 2,above: n = 2), ERO(below: n = 14, above: n = 1), regurgitant volume (below: n = 14, above: n = 1).

**Table 3:** Left heart assessments of patients delineated by proportionate or disproportionate TR.

	Tricuspid Regurgitation		
Left Heart Parameters	Proportionate TR (VC/TA < 0.24 mL) n = 224	Disproportionate TR (VC/TA ≥ 0.24 mL) n = 132	*p* Value
Left atrium			
LA size (cm^2^)	27.5 (23.2–32.8)	29.3 (25.6–34.6)	0.015
Indexed LA size (cm^2^/m^2^)	14.2 (11.9, 17.1)	16.1 (13.9, 19.2)	<0.001
Left ventricle volumes			
LVEDV (mL)	101 (75.0–138)	93.0 (62.0–143)	0.20
Indexed LVEDV (mL/m^2^)	53.5 (37.3–71.2)	51.9 (36.6–73.2)	0.65
LVESV (mL)	59.5 (35.3–87.0)	49.0 (28.8–90.3)	0.20
Indexed LVESV (mL/m^2^)	30.3 (18.3–46.0)	27.7 (18.3–46.0)	0.63
Left ventricle performance			
LV ejection fraction (%)	43.0 (32.0–56.0)	45.0 (33.0–56.0)	0.63
LV dP/dt (mmHg)	928 (707–1242)	855 (662–1218)	0.65
S’ (cm/s)	0.07 (0.06–0.09)	0.07 (0.06–0.10)	0.70
Left ventricular strain			
GLS (%)	−9.48 (−12.4–−6.36)	−9.10 (−12.9–−5.82)	0.69
GLSR	−0.67 (−0.84–−0.48)	−0.66 (−0.91–−0.47)	0.63
>Mild Mitral Regurgitation	36.6	51.5	0.0059

Abbreviations: left atrium (LA), left ventricular end-diastolic volume (LVEDV), LV end-systolic volume (LVESV), tissue Doppler imaging lateral mitral annular S wave velocity (S’), global longitudinal strain (GLS), GLS rate (GLSR). All data are presented as median (IQR) and between group differences were evaluated using the non-parametric Mann–Whitney U test. Patient data were missing for follow parameters (above/below threshold). LVEDV (below = 2), LVESV (below: n = 2), LV ejection fraction (below: n = 2), LV dp/dt (below: n = 35, above: n = 13), S’ (below: n = 4, above: n = 2), GLS (below: n = 2, above: n = 1).

**Table 4:** Kaplan–Meier estimates for overall survival at 2 years for 4 different quantitative assessments of significant or disproportionate TR.

		Mild	Moderate	Severe
Regurgitant volume (TR RegVol)	Number at risk	171	43	130
	Mortality *n (%)*	65 (38.0)	19 (44.2)	51 (39.2)
	Hazard ratio (mild vs. moderate) [95% Cis]	0.790 [0.458–1.364]		
	*p*-value (Logrank)	0.400		
	Hazard ratio (mild vs. severe) [95% Cis]	0.937 [0.652–1.348]		
	*p*-value (Logrank)	0.700		
	Hazard ratio (moderate vs. severe) [95% Cis]	1.186 [0.675–2.086]		
	*p*-value (Logrank)	0.500		
	*p*-value overall (Logrank)	0.661		
Effective Regurgitant orifice (TR ERO)	Number at risk, n	84	91	169
	Mortality, n (%)	29 (34.5)	40 (44.0)	66 (39.1)
	Hazard ratio (mild vs. moderate) [95% Cis]	0.722 [0.451–1.154]		
	*p*-value (Logrank)	0.200		
	Hazard ratio (mild vs. severe) [95% Cis]	0.820 [0.544–1.237]		
	*p*-value (Logrank)	0.400		
	Hazard ratio (moderate vs. severe) [95% Cis]	1.136 [0.753–1.715]		
	*p*-value (Logrank)	0.500		
	*p*-value overall (Logrank)	0.404		
Vena contracta (TR VCD)	Number at risk	19	154	181
	Mortality *n (%)*	7 (36.8)	60 (39.0)	76 (42.0)
	Hazard ratio (mild vs. moderate) [95% Cis]	0.872 [0.426–1.784]		
	*p*-value (Logrank)	0.700		
	Hazard ratio (mild vs. severe) [95% Cis]	0.768 [0.377–1.564]		
	*p*-value (Logrank)	0.500		
	Hazard ratio (moderate vs. severe) [95% Cis]	0.881 [0.628–1.236]		
	*p*-value (Logrank)	0.500		
	*p*-value overall (Logrank)	0.659		

The mild, moderate and severe estimates are threshold values of RegVol, ERO and VC diameter used to subcategorise patient clinical features. The values vary depending on the variable in question: RegVol mild, moderate and severe thresholds were determined at <30, 30–44 and ≥45, ERO mild, moderate and severe thresholds were determined at values of <0.3, 0.3–0.69 and ≥ 0.70, respectively and VC diameter mild, moderate and severe thresholds were determined at <0.2, 0.2–0.39 and ≥0.40, respectively. As there were values missing for mortality data (below: n = 2, above: n = 2) the corresponding data from each metric were excluded for analysis. Additionally, data were missing for the ERO(below: n = 14, above: n = 1) and regurgitant volume (below: n = 14, above: n = 1) due to inability to accurately measure PISA radii.

**Table 5:** Unstratified Kaplan–Meier estimates for overall survival at 2 years of significant or disproportionate TR.

		Below (VC/TA ≤ 0.24) (n = 224)	Above (VC/TA ≥ 0.24) (n = 132)
Disproportionate TR	Number at risk, n	222	130
	Mortality, n (%)	81 (36.5)	62 (47.7)
	Hazard ratio (below vs. above) [95% Cis]	1.48 [1.06–2.06]	
	*p*-value (Logrank)	0.020	

As there were values missing for mortality data (below: n = 2, above: n = 2) the corresponding data from the disproportionate TR metric were excluded for analysis.

**Table 6:** Multivariate analysis of the full cohort.

Variable	B	SE	Wald	Exp(b)	95% CIs	*p*
Age (n = 414)	0.04425	0.01134	15.2307	1.0452	1.0223–1.0687	0.0001
BMI (n = 411)	−0.003826	0.01326	0.08328	0.9962	0.9706–1.0224	0.7729
CKD (n = 414)	0.2744	0.2718	1.0187	1.3157	0.7723–2.2415	0.3128
COPD (n = 414)	0.7765	0.2182	12.6595	2.1738	1.4173–3.3342	0.0004
Moderate + MR (n = 414)	0.2667	0.1887	1.9975	1.3057	0.9020–1.8900	0.1576
SPAP (n = 401)	0.009458	0.004840	3.8187	1.0095	1.0000–1.0191	0.0507
LVEDV (n = 411)	−0.004859	0.002217	4.8047	0.9952	0.9908–0.9995	0.0284
Urea (n = 414)	0.04250	0.02049	4.3023	1.0434	1.0023–1.0862	0.0381
Sodium (n = 414)	−0.02667	0.01586	2.8291	0.9737	0.9439–1.0044	0.0926
BNP (n = 414)	0.0002427	0.00006974	12.1076	1.0002	1.0001–1.0004	0.0005
Hb (n = 414)	−0.0004978	0.004365	0.01301	0.9995	0.9910–1.0081	0.9092
eGFR (n = 413)	0.001103	0.01188	0.008629	1.0011	0.9781–1.0247	0.9260
Disproportionate TR (n = 352)	−0.01264	0.1842	0.004707	0.9874	0.6882–1.4168	0.9453

## Data Availability

Data will be made with a signed data access agreement for 6 months after the publication of this manuscript.

## Supplementary Materials

The following supporting information can be downloaded at: https://www.mdpi.com/article/10.3390/diagnostics13010109/s1, File S1: Echocardiographic assessment protocol; File S2: Flow diagram for participant exclusion; Table S1: Kaplan-Meier estimates of mild vs. >mild TR; Table S2: Kaplan-Meier estimates of non-severe vs. severe TR; Table S3: Univariate analysis of the full cohort; Table S4: Univariate analysis of the Fortuni cohort; Table S5: Multivariate analysis of the Fortuni cohort.
