# Supplementary material for: Tricuspid Regurgitation in Acute Heart Failure: Predicting Outcome Using Novel Quantitative Echocardiography Techniques
Source: Diagnostics (Basel). 2022 Dec 29;13(1):109. doi: 10.3390/diagnostics13010109 (PMC9818460; doi:10.3390/diagnostics13010109)
Supplement: Supplementary file 1 [file diagnostics-13-00109-s001.zip › diagnostics-2079391-supplementary.pdf]

Supplementary File S1

Echocardiographic assessment protocol.

Views and acquisition requirements

(2 cardiac cycles in sinus rhythm, 5 cardiac cycles in atrial fibrillation)

1. *Parasternal Long Axis view:*

• 2D loop at moderate (50-70/sec) frame rate
• 2D loop with colour Doppler
• 2D loop with colour tissue Doppler at high (>120/sec) frame rate
• M-mode (still frame) of aortic root/left atrium
• M-mode (still frame) of left ventricle
• 2D loop (zoomed) of aortic root for Left-ventricular outflow tract dimension
• 2D loop RV inflow view (optional)
• 2D loop RV inflow view with colour wave Doppler for TR (optional)

2. *Parasternal Short Axis view:*

• 2D loop at aortic valve level
• Pulse wave Doppler of right ventricular outflow tract (sweep)
• 2D loop of MV in short axis
• 2D loop with colour Doppler at MV level at high frame rate
• 2D loop at papillary muscle level at moderate frame rate
• 2D loop with colour tissue Doppler at high (>120/sec) frame rate
• 2D loop at mid left ventricular level
• 2D loop at apical left ventricular level

3. *Apical Four Chamber View:*

• 2D loop at moderate frame rate focusing on all 4 chambers
• 2D loop with colour flow Doppler on all 4 chambers
• 2D loop zoom at moderate frame rate focusing on left and right ventricles
• 2D loop zoom with colour tissue Doppler at high frame rate focusing on left and right ventricles
• Pulsed tissue Doppler of the lateral mitral annulus.
• Pulsed tissue Doppler of the septal mitral annulus
• Pulsed tissue Doppler of the right ventricular free wall annulus
• 2D loop with colour flow Doppler focusing on MR (must see proximal isovelocity surface area (PISA), vena contracta (VC) and jet in left atrium)
• Zoomed 2D loop with colour flow Doppler focusing on MR (must see PISA, VC and jet in LA)
• 2D loop with colour flow Doppler focusing on tricuspid regurgitation (TR)
• 2D loop at high frame rate to include both atria and pulmonary veins
• Pulsed Doppler of the mitral inflow (sweep)

• Pulsed wave Doppler of the right upper pulmonary vein (sweep)
• Continuous wave Doppler of the MR jet
• Pulsed Doppler of the left ventricular outflow (sweep)
• Continuous wave Doppler of aortic outflow
• Pulsed Doppler of the tricuspid inflow (sweep)
• Continuous wave Doppler of the TR jet

4. *Apical Two Chamber view:*

• 2D loop at moderate frame rate (LA/LV)
• 2D loop with colour flow Doppler
• 2D loop at moderate frame rate focusing on left ventricle
• 2D loop with colour tissue Doppler at high frame rate focusing on left ventricle
• 2D loop zoom at high frame rate on left atrium and pulmonary veins
• 2D loop with colour flow Doppler focusing on MR (must see PISA, VC and jet in LA)
• Zoomed 2D loop with colour flow Doppler focusing on MR (must see PISA, VC and jet in LA)

5. *Apical Long Axis view:*

• 2D loop at moderate frame rate
• 2D loop at moderate frame rate focusing on left ventricle
• 2D loop with colour Doppler
• 2D loop with colour flow Doppler focusing on MR (must see PISA, VC and jet in LA)
• Zoomed 2D loop with colour flow Doppler focusing on MR (must see PISA, VC and jet in LA)

6. *Subcostal view:*

• Inferior vena cava dimension and sniff test – 2D ± M-mode
• 2D loop of Subcostal long axis view
• 2D loop of Subcostal short axis view at papillary muscle level (optional)
• 2D loop of Subcostal short axis view at atrioventricular level (optional)

Supplementary File S2; Flow diagram for participant exclusion

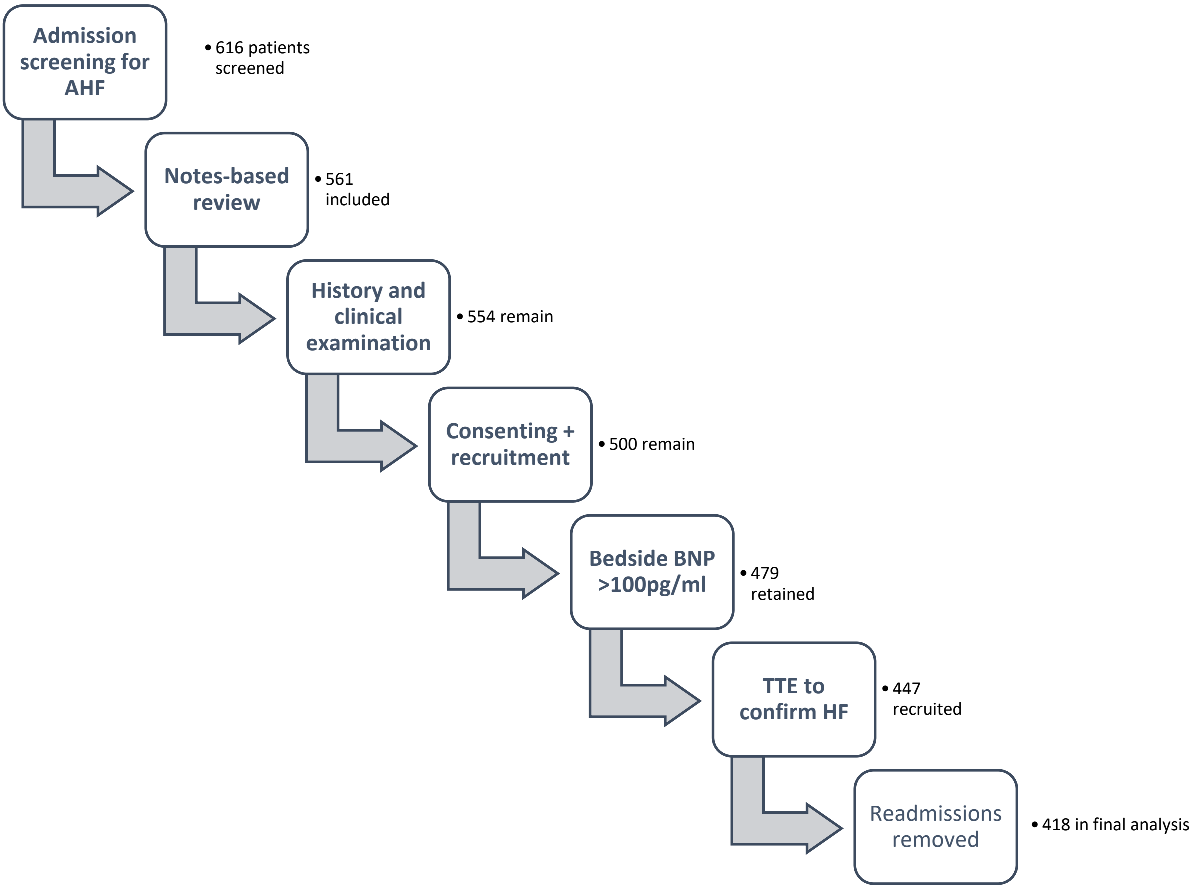
Admission screening for AHF
• 616 patients screened
Notes-based review
• 561 included
History and clinical examination
• 554 remain
Consenting + recruitment
• 500 remain
Bedside BNP >100pg/ml
• 479 retained
TTE to confirm HF
• 447 recruited
Readmissions removed
• 418 in final analysis

Supplementary Table S1: Kaplan-Meier estimates of mild vs. >mild TR

		Mild TR		Haemodynamically Significant TR (>mild)
**Regurgitant volume**				
	Number at risk, n	171		173
	Mortality, n	73		70
	Hazard ratio (significant vs mild) [95% CIs]		1.10 [0.78,1.54]	
	p-value (Logrank)		0.58	
**Effective Regurgitant orifice**				
	Number at risk, n	84		260
	Mortality, n	29		106
	Hazard ratio (significant vs mild) [95% CIs]		1.25[0.85,1.84]	
	p-value (Logrank)		0.26	
**Vena contracta**				
	Number at risk, n	19		335
	Mortality, n	7		135
	Hazard ratio (significant vs mild) [95% CIs]		1.20[0.58,2.41]	
	p-value (Logrank)		0.61	

Supplementary Table S2: Kaplan-Meier estimates of non-severe vs. severe TR

		Non-Severe TR (≤moderate)		Severe TR
**Regurgitant volume**				
	Number at risk, n	214		130
	Mortality, n	84		51
	Hazard ratio (severe vs non-severe) [95% CIs]		1.03 [0.72,1.46]	
	p-value (Logrank)		0.87	
**Effective Regurgitant orifice**				
	Number at risk, n	175		169
	Mortality, n	69		66
	Hazard ratio (severe vs non-severe) [95% CIs]		1.01[0.72,1.41]	
	p-value (Logrank)		0.97	
**Vena contracta**				
	Number at risk, n	173		181
	Mortality, n	67		76
	Hazard ratio (severe vs. non-severe) [95% CIs]		1.14[0.82,1.58]	
	p-value (Logrank)		0.44	

Supplementary Table S3. Univariate analysis of the full cohort

Variable	Hazard Ratio (95% CIs)	p *
Age (n=414)	1.056(1.038-1.074)	<0.0001*
Gender (n=414)	1.093(0.802-1.489)	0.574
BMI (n=411)	0.970(0.949-0.992)	0.0077*
Coronary artery disease (n=414)	1.240(0.905-1.699)	0.181
Hypertension (n=414)	1.286(0.938-1.763)	0.119
Diabetes (n=414)	1.141(0.8224-1.582)	0.430
CKD (n=414)	1.931(1.411-2.641)	<0.0001*
COPD (n=414)	1.924(1.322-2.800)	0.0006*
Previous CVA (n=414)	0.861(0.549-1.351)	0.516
Right atrial size (n=412)	1.006(0.988-1.023)	0.543
Left atrial size (n=414)	1.001(0.991-1.028)	0.313
Moderate+MR (n=414)	1.396(1.023-1.903)	0.0354*
SPAP (n=401)	1.012(1.012-1.028)	<0.0001*
TAPSE (n=411)	0.793(0.578-1.090)	0.153
LVEF (n=411)	1.002(0.992-1.013)	0.699
LVEDV (n=411)	0.997(0.994-0.100)	0.0314*
Urea (n=414)	1.060(1.040-1.082)	<0.0001*
Sodium (n=414)	0.947(0.923-0.973)	0.0001*
BNP (n=414)	1.0003(1.0002-1.0004)	<0.0001*
Hb (n=414)	0.990(0.984-0.997)	0.004*
eGFR (n=413)	0.977(0.967-0.987)	<0.0001*
RVA diastole index (n=383)	1.042(0.998-1.087)	0.059
Disproportionate TR (n=352)	1.481 (1.064-2.061)	0.020*

Data was analysed using a cox-proportional hazards model to generate survival estimates between the subgroups of each variable.

Supplementary Table S4. Univariate analysis of the Fortuni cohort

Variable	Hazard Ratio (95% CIs)	p *
Age (n=59)	1.028(0.981-1.077)	0.236
Gender (n=59)	1.135(0.501-2.568)	0.762
BMI (n=59)	0.976(0.936-1.017)	0.247
Coronary artery disease (n=59)	0.631(0.237-1.681)	0.357
Hypertension (n=59)	0.729(0.331-1.605)	0.432
Diabetes (n=59)	0.990(0.445-2.205)	0.981
Chronic kidney disease (n=59)	3.055(1.313-7.108)	0.00953*
COPD (n=59)	3.067(1.316-7.147)	0.0094*
Previous CVA (n=59)	0.290(0.068-1.232)	0.0935
Right atrial size (n=59)	1.043(1.010-1.079)	0.0116*
Left atrial size (n=59)	1.026(0.991-1.063)	0.141
Moderate+MR (n=59)	0.871(0.364-2.085)	0.756
SPAP (n=59)	1.023(1.003-1.043)	0.0252*
TAPSE (n=59)	0.848(0.384-1.873)	0.684
LVEF (n=59)	1.015(0.943-1.093)	0.686
LVEDV (n=59)	0.997(0.985-1.010)	0.689
Urea (n=59)	1.154(1.079-1.236)	<0.0001*
Sodium (n=59)	0.971(0.910-1.035)	0.365
BNP (n=59)	1.000(1.000-1.001)	0.0782
Hb (n=59)	0.984(0.963-1.006)	0.157
eGFR (n=59)	0.967(0.945-0.990)	0.00525*
RVA diastole index (n=59)	1.060(0.991-1.135)	0.094
Disproportionate TR (n=59)	2.034(0.869-4.751)	0.102

Data was analysed using a cox-proportional hazards model to generate survival estimates between the subgroups of each variable. The Fortuni cohort is a subgroup of patients who had greater than mild TR and an LVEF value of ≥ 55.

Supplementary Table S5. Multivariate analysis of the Fortuni cohort

Variable	B	Se	Wald	Exp b	95% CI	p*
Chronic Kidney disease (n=59)	0.863	0.632	1.365	2.370	0.686-8.188	0.172
COPD (n=59)	1.286	0.529	2.429	3.618	1.282-10.21	0.015*
Right atrial size (n=59)	0.031	0.018	1.754	1.032	0.996-1.069	0.079
SPAP (n=59)	0.012	0.012	1.025	1.012	0.989-1.036	0.305
Urea (n=59)	0.151	0.060	2.558	1.163	1.036-1.305	0.011*
eGFR (n=59)	0.018	0.018	0.997	1.018	0.983-1.055	0.319

Data was analysed using a cox-proportional hazards model to generate survival estimates between subgroups of each variable and with respect to the covariates selected. The covariates listed were selected for posthoc, multivariate analysis if demonstrating statistical significance in the univariate analysis reported in table 3. The Fortuni cohort is a subgroup of patients who greater than mild TR and an LVEF value of ≥ 55.
